# Characterization, Functional Properties, and Resistant Starch of Freshwater Macrophytes

**DOI:** 10.1155/2021/8825970

**Published:** 2021-01-21

**Authors:** Farahin N. N. Syed, Muta H. Zakaria, Japar S. Bujang, Annie Christianus

**Affiliations:** ^1^Department of Aquaculture, Faculty of Agriculture, Universiti Putra Malaysia, 43400 UPM Serdang, Selangor Darul Ehsan, Malaysia; ^2^International Institute of Aquaculture and Aquatic Sciences (i-AQUAS), Universiti Putra Malaysia, 71050 Port Dickson, Negeri Sembilan, Malaysia; ^3^Department of Biology, Faculty of Science, Universiti Putra Malaysia, 43400 UPM Serdang, Selangor Darul Ehsan, Malaysia

## Abstract

Several aquatic macrophytes such as *Colocasia esculenta*, *Eleocharis dulcis*, *Nelumbo nucifera*, *Sagittaria sagittifolia*, *Trapa bispinosa*, and *Typha angustifolia* possessed carbohydrate mainly in their storage and reproductive parts. Starch morphology, total starch, and amylose content of these six freshwater plant species were determined. Their functional properties, i.e., starch crystallinity, thermal properties, and rheological behaviour were assessed. Large starch granules were in *N. nucifera* rhizome (>15 *μ*m), medium-sized was *N. nucifera* seed (8-18 *μ*m), while the rest of the starches were small starch granules (<8 *μ*m). Shapes of the starch granules varied from oval and irregular with centric hilum to elongated granules with the eccentric hilum. *Eleocharis dulcis* corm starch had significantly higher total starch content (90.87%), followed by corms of *C. esculenta* (82.35%) and *S. sagittifolia* (71.71%). *Nelumbo nucifera* seed starch had significantly higher amylose content (71.45%), followed by *T. angustifolia pollen* (36.47%). In comparison, the waxy starch was in *N. nucifera* rhizome (7.63%), *T. bispinosa* seed (8.83%), *C. esculenta* corm (10.61%), and *T. angustifolia* rhizome (13.51%). Higher resistant starch was observed mostly in rhizomes of *N. nucifera* (39.34%)>*T. angustifolia* (37.19%) and corm parts of *E. dulcis* (37.41%)>*S. sagittifolia* (35.09%) compared to seed and pollen starches. The XRD profiles of macrophytes starches displayed in all the corms and *N. nucifera* seed had A-type crystallinity. The *T. bispinosa* seed had C_A_-type, whereas the rest of the starches exhibited C_B_-type crystallinity. Waxy starches of *C. esculenta* corm had higher relative crystallinity (36.91%) and viscosity (46.2 mPa s) than regular starches. Based on thermal properties, high-amylose of *N. nucifera* seed and *T. angustifolia* pollen resulted in higher gelatinization enthalpy (19.93 and 18.66 J g^−1^, respectively). Starch properties showed equally good potential as commercial starches in starch-based food production based on their starch properties and functionality.

## 1. Introduction

Starch plays a vital role in food and nonfood industries, e.g., pharmaceutical, paper, textiles, biomedical, and polymer, because of its gelling characteristics, thickening, water binder, and food system stabilizing capacities [1]. Research on the structure and physicochemical properties of starch in cultivated plants, *Zea mays* (maize), *Manihot esculenta* (cassava), and *Solanum tuberosum* (potato) resulted in their extensive utilization in food industries. However, other plants besides those mentioned above may possess potential and promising alternative starch sources.

Over the years, aquatic plants' usage has become increasingly important; for example, rice, *Oryza sativa*, is a human staple diet [2]. Detailed studies of starch isolated from aquatic macrophytes are increasing and mostly focused on specific plants such as water chestnut, lotus, rice, and taro. Asian countries such as China and Japan had cultivated aquatic macrophytes such as lotus (*Nelumbo nucifera*), Chinese water chestnut (*Eleocharis dulcis*), water caltrop (*Trapa bispinosa*), taro (*Colocasia esculenta*), and arrowhead (*Sagittaria* sp.) for starch-based food. The research conducted showed that starches from macrophytes could also be a promising candidate as an energy source in the food-related industry. Water chestnut corm flour as a thickening agent and dusting powder in food preparation [3], arrowhead corms, and water caltrop fruits are eaten boiled or cooked and can be dried and ground into a powder [4, 5]. Taro tuber possesses low fat, high carbohydrate, and minerals content and suitable as a food ingredient for baby food, chips, and bread [6]. Lotus seeds, consumed boiled or processed into powder, are also used in the pharmaceutical industry to treat inflammation, arrhythmia, cancer, and skin diseases [7]. Most of the starch's diverse uses are from cultivated species [8–11], and research on the wild species is still scarce [12].

Research on starch isolated from freshwater macrophytes such as cattails, arrowhead, yellow nutsedge, and duckweed and their physicochemical properties are also available and less prevalent [13, 14]. Although consumed by local communities, their local utilization was seldom reported in the literature. Nowadays, consumers are engaging with resistant starch (RS) to promote health benefits similar to high-amylose starch. RS is poorly digested starches and absorbed in healthy individuals' small intestine due to its complex molecular structure [15]. They are either entirely or partially fermented as a food source for bacteria, primarily inhabiting the colon. There are limited studies conducted regarding RS in aquatic macrophytes starches.

Investigating the aquatic macrophytes starches, among others, is to create awareness of their various uses and economic values. For those involved in aquatic macrophytes, it can be part of their added income generation. This study also investigated the potential use of aquatic macrophytes starches in other applications in a starch-based industry. Thus, the present study was to systematically evaluate the starch structure, composition, functional properties, and also their resistant starch (RS) content isolated from selected commonly consumed aquatic macrophytes such as taro, lotus, and water chestnut and rarely consumed, e.g., arrowhead, water caltrop, and cattail in Malaysia.

## 2. Materials and Methods

### 2.1. Plant Materials

Two kilograms (2) kg of edible storage organ from five macrophytes species; corms of *E. dulcis*, *S. sagittifolia*, and *C. esculenta*; rhizomes of *N. nucifera* and *T. angustifolia*; seeds of *N. nucifera* and *T. bispinosa*; and pollen of *T. angustifolia* were peeled, washed, and isolated for starches.

### 2.2. Isolation of Native Starch

Native starch was isolated following a method described by Vasanthan [16] with a slight modification. The plant materials were added to water in a ratio of 1 : 10. The mixture was then blended for 5-10 minutes until a smooth slurry is formed. Approximately 0.01% (*w*/*v*) sodium metabisulfite was added into the slurry and left for 30 minutes before filtering using 100 *μ*m nylon mesh cloth. The filtrated starch was centrifuged at 8000 rpm at 20°C for 20 minutes. The supernatant was discarded, and the pellet was oven-dried at 40°C for 24 hours. The dried starch was ground using mortar and pestle, sieved (250 *μ*m), labelled, stored in a tightly closed container, and kept dry in a desiccator (10% relative humidity).

### 2.3. Polarized Optical Microscopy

A small amount (0.2 mg) of starch powder was placed on a microscope slide (25.4 mm × 76.2 mm) by using a spatula. The starch was stained with 0.25% Lugol's solution. The slide was then covered with a coverslip and observed under a compound light microscope (DM 750, Leica Microsystem, Wetzlar, Germany) equipped with a camera set (ICC50 W, Leica Microsystem, Wetzlar, Germany), polarized filter and analyzer. Images of starch granule and hilum were observed and captured. The granule sizes were measured using the ImageJ software (NIH, US).

### 2.4. Scanning Electron Microscopy (SEM)

Structural characteristics of the starch granules were examined with scanning electron microscope Jeol JSM-6400 (Jeol Ltd., Tokyo, Japan) and analyzed with an energy dispersive X-ray analyzer (EDS) PGT Spirit at an acceleration of 20 keV. Samples of starch were mounted on aluminium specimen stubs with double-sided adhesive tape and sputtered with a 20-30 nm gold layer using a sputter coater before observation.

### 2.5. Chemical Properties and Resistant Starch

Macrocomponents (total starch and amylose) and resistant starch were determined using the Megazyme assay kit with given procedures (Megazyme International Ireland Ltd., Bray, Ireland). Microcomponents, i.e., protein, lipid, and phosphorus, were determined the content by following the Official Method of AOAC International [17].

### 2.6. X-Ray Diffraction

Starch powders were scanned through the 2*θ* of 5°-45° using X-ray diffractograms (Xpert Pro MPD, Philips, Netherlands). Traces were obtained using a Cu-K*α* radiation detector with a nickel filter and scintillation counter operating under the following conditions: 40 kV, 30 mA, scattering slit 25 nm, K-Alpha1 wavelength 1.78901 Å, K-Alpha1 wavelength 1.7929 Å, Ratio K-Alpha2/K-Alpha1 0.5, and scanning rate of 0.02°/min. The degree of crystallinity of samples was estimated and analyzed following the method of Zhang et al. [15].

### 2.7. Starch Gelatinization

Thermal properties of starches were studied using differential scanning calorimeter, DSC (Model-823e, Mettler-Toledo, Switzerland). Starch (~10 mg, dry weight) was placed into a 40 *μ*L capacity aluminium pan with the addition of 70% distilled water to achieve starch-water suspension. The DSC analyzer's calibration was conducted using indium, and an empty aluminium pan was used as a reference. Sample pans were heated from 25 to 120°C at the rate of 10°C/min. Onset temperature (To), peak temperature (*Tp*), conclusion temperature (*Tc*), and gelatinization enthalpy (Δ*H*) (J/g dry starch) were determined in triplicate.

### 2.8. Rheological Behaviour

Rheological properties of starches suspended in distilled water were determined by rotational rheometer (C-DG26.7/QC, RheolabQC, Anton Par Ltd, Germany). 6% (*w*/*v*) suspension of native starches were prepared by dispersing a suitable mass of dried starch granules in distilled water by a ratio of 1 : 17 with constant stirring. The viscosity (mPa s) and shear stress (Pa) were determined following the method by Chrungoo and Devi [18].

### 2.9. Statistical Analysis

The data recorded in all the tables were mean values and standard error. Analysis of variance (1-way ANOVA) was performed for the data and, if significant, followed by a post hoc Duncan's multiple range test (DMRT) (*p* < 0.05) using the SPSS 16.0 Statistical Software Program, IBM, Chicago, IL.

## 3. Results and Discussion

### 3.1. Starch Granule Morphology

**Figure 1:**
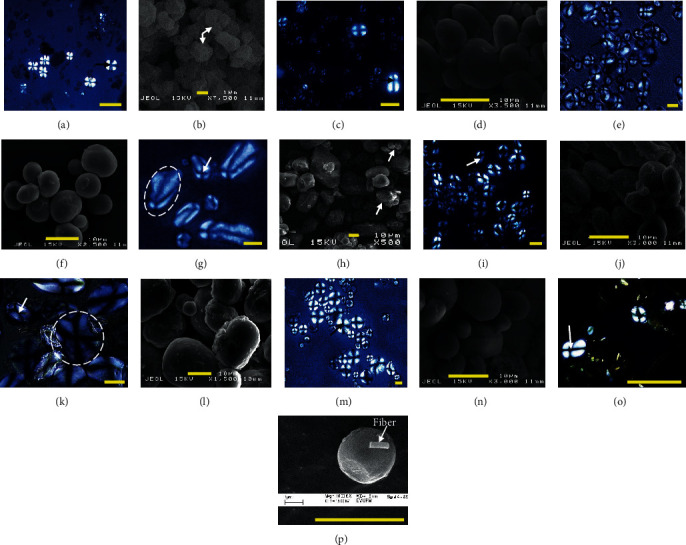
Starch granules of aquatic macrophytes captured (a–h) under polarized microscope, 40X magnification and (b, d, f, h), under scanning electron microscope: (a, b) *Colocasia esculenta* corm, (c, d) *Eleocharis dulcis* corm, (e, f) *Sagittaria sagittifolia* corm, (g, h) *Nelumbo nucifera* rhizome, (i, j) *Nelumbo nucifera* seed, (k, l) *Trapa bispinosa* seed, (m, n) *Typha angustifolia* rhizome, and (o, p) *Typha angustifolia* seed. The yellow bar scale represents 10 *μ*m length and 1 *μ*m length for a, c, e, g and b, d, f, h, respectively. Arrows in the polarized image indicate the hilum (Maltese cross).

Starch granules of plant species varied in size from 1 mm up to 100 mm, for taxonomic discrimination to be possible. *Nelumbo nucifera* rhizome had significantly larger starch granules with 20.96 *μ*m. In contrast, the smaller granules were in *C. esculenta* corm and *T. angustifolia* pollen with 2.95 and 2.09 *μ*m, respectively (Table 1). Pomeranz [19] categorized the starch granule size based on commercial starch into three groups; large, 15-100 *μ*m (potato starch), medium-sized, 10-25 *μ*m (maize or corn starch), and small, 3-8 *μ*m (rice starch). From the starch classification, *N. nucifera* rhizome possesses large starch granules while *N. nucifera* seed has medium-sized starch granules that ranged 8.11-17.78 *μ*m. The corms of *C. esculenta, E. dulcis* and *S. sagittifolia*, *T. bispinosa* seed, *T. angustifolia* rhizome, and pollen have small starch granules ranging 0.4-13.44 *μ*m. The size of starch granules affects starch granules gels and paste performance as the larger the granule, the faster it swells, due to less molecular bonding than smaller granule [20]. For example, potato starch possessed a large granule (15-100 *μ*m), which resulted in faster gelatinization range (56-69°C). In contrast, the smaller granules of regular corn (5-25 *μ*m) resulted in a slightly slower gelatinization range with 62-80°C (Pomeranz, 2019). In this present study, a large starch granule of *N. nucifera* rhizome gelatinizes faster than others. Besides, Pomeranz [19] also reported that small starch granules are relatively rare, which are suitable in dusting starches used in candy dusting, cosmetics, filling agent for the biodegradable polyethylene film, and tyre molding release agents. Also, in taro, its small starch has been proven to be easily digested, hence a potential commercial value in baby foods and patients with gastrointestinal problems for ease of bioassimilation [21]. The granular structure and shape were also varied, as shown in Figure 1. Small granules of *C. esculenta* corm and *T. angustifolia* had predominantly polygonal and irregular shapes with few oval shapes. In contrast, the larger granules normally were observed with predominantly longitudinal and rod-shaped such as in *N. nucifera* rhizome. Tester et al. [22] reported that starch granules were as simple or compound. Some plant contains compound granules (*C. esculenta* corm and *N. nucifera* rhizome) due to the fusing of different granules developing simultaneously within a single amyloplast during biosynthesis [23]. The rest of the species were as simple granules.

Besides, some starch granules may still attach to other nonstarch components like fiber, lipid, or protein fragments despite undergoing isolating process, as shown in *T. angustifolia* seed starch (Figure 1(h)). According to Svihus et al. [24], these components, especially lipid and protein associated with granules, may influence digestion by reducing the contact between starch and digestive enzymes, thus slowing the swelling of the granules and gelatinization process. The position of the hilum or Maltese cross was also considered one of the unique identifications as it described the nucleus around which the starch form [25]. All the aquatic macrophytes starch samples showed distinct Maltese cross across the hilum when viewed under a polarized microscope. Round- and oval-shaped granules were with centric hilum, such as in corm of *C. esculenta* and *E. dulcis*, and rhizome of *T. angustifolia*. On the other hand, hilum was observed eccentrically at the small end of the elongated granules of *S. sagittifolia* corm and *N. nucifera* rhizome. Large granule like potato starch displayed pronounced striations around the central hilum where it is the botanical point of origin for the granule to grow [19], which can be seen in the rhizome of *N. nucifera* and the seed of *T. bispinosa* (circled granules) even though the layer not as distinct as reported in potato.

### 3.2. Chemical Properties

The starch paste behaviour and functionality depend on the composition of amylose and amylopectin and the microcomponent as such affects the final food quality.  Table 2 shows that the moisture content of the starch flours from the present study is in the range between 8.91% of *T. angustifolia* pollen and 17.23% of *E. dulcis* corm starch. A higher total starch was in corm samples, including *E. dulcis* (90.87%), *C. esculenta* (82.35%), and *S. sagittifolia* (71.71%), whereas the *T. angustifolia* pollen has a significantly least starch content (7.02%). Based on previous studies, *Typha* sp. generally had low starch contents ranging between 13.01% and 14.5% compared to other commercial starch such as *Zea mays* (22.40%) [26, 27]. The composition of amylose and amylopectin gives variable effects of starch swelling capacity, water solubility, water-binding capacity, and microscopic characteristics, hence carried different industrial applications [28]. Normal starch generally contains 75% of amylopectin and 25% of amylose, while waxy starch contains <15% amylose and 20-35% amylopectin. Regular starch and high-amylose starches contain >40% amylose [22]. The present study shows that *N. nucifera* seed had high-amylose content with 71.45%, whereas *T. angustifolia* pollen and *S. sagittifolia* corm were categorized as regular starches with an amylose content of 36.47% and 19.80%, respectively. The rest were classified as waxy starch, ranging from 7.63% in *N. nucifera* rhizome to 13.51% in *T. angustifolia* rhizome. According to Eltaboni et al. [29], starch granules may contain amylose up to 80% as observed in the *N. nucifera* seed starch of the present study. In addition, a wide range of amylose content was observed in 46 lotus roots starches from 2.31% of Lianhu wild lotus to 60.52% of Jianxuan-35 hao lotus root [30]. A starch with high-amylose content usually had a small and narrow size range with nonsymmetrically shape granules [31]. Similarly, such characteristics of starch granules of *N. nucifera* seed and *T. angustifolia* pollen also possess a higher amylose content. The high amylose of *N. nucifera* seed starch can be applied as a gelling agent or in the biodegradable plastic film industry. The chemical properties of the less-branched amylose molecule also provide beneficial effects to human health. Meals prepared with high amylose slow the rate of digestion, as it possesses the higher crystallinity which generated a postprandial glucose response in the attributed blood and lessened the fasting concentration of triacylglycerol and cholesterol [32]. Consequently, high-amylose *N. nucifera* seed starch may reduce the blood glucose and insulin level and enhance the body's fat burning.

In contrast, waxy starch with higher amylopectin which possesses branched and long chain length greater than 30 chains yielded higher molecular weight. Thus, this branched chain hindered each particle from reassociating and formed an ordered structure that resulted in weaker hydrogen bonding and the strength of the gel [20]. Amylopectin with the clustered structures reduced retrogradation, gelation, and syneresis (loss of water). However, they are better at building viscosity and the production of more slimy paste. This suggested that waxy starch in this study are suitable in making soft-textured food such as dessert-like products, baby foods, gravies, sauces, and thickening soup due to its excellent water retention properties, porous texture, and stickiness criteria [33]. Generally, foods with higher amylopectin have a higher glycemic index as they are readily digestible and absorbed after consumed. Thus, it is advisable to opt for food lower in amylopectin to prevent the raising of blood sugar, insulin, and cholesterol levels. However, recent studies show that scientists have devised various ways to minimise food digestibility for waxy starches via an enzymatic process, encapsulation, extrusion cooking, recrystallization, and starch chemical modification [33]. Under this new development, the modified waxy starches such as wheat, rice, and maize have great potential as novel functional food ingredients, which also equally applied for waxy aquatic macrophytes starches.

The minor components analysis showed higher protein accumulated in *T. bispinosa* seed (26.47%) followed by *T. angustifolia* pollen (15.70%) starches, whereas the least was from *S. sagittifolia* corm with 3.06%. Higher lipid contents were in *T. angustifolia* rhizome and pollen (3.87% and 3.23%, respectively) and *N. nucifera* seed (2.96%). According to Tester et al. [22], proteins and lipids can limit the starch functionality as they can associate with starch granule on the surface and internal components. Protein contents also influenced the pasting activity as higher protein will build up the interaction's binding forces, thus reducing the pasting temperature [34]. The presence of flexible protein molecules may reduce the water's surface tension and increase the foaming tendency, which is important in maintaining the structure and texture of food products such as baked goods and ice cream [34]. However, higher protein may also have undesired side effects. It can produce browning and off-flavor in foods due to its positive reaction with major food components such as reducing sugars, polyphenols, fats, and other oxidation products [25]. Svihus et al. [24] reported that smaller granules usually have higher lipid contents, which are also observed in small granules of *T. angustifolia* pollen and rhizome. Starch-lipid compositions used in several applications, such as stabilizers and thickener agents, reduced starch stickiness, dough conditioners, enhanced freeze-thaw stability, detain bread, and biscuit staling and bread crumb softener [35].

### 3.3. Resistant and Nonresistant Starch

Table 3 represents resistant (RS) and nonresistant starch (non-RS) content in the freshwater macrophytes starch samples in comparison with previous studies. Higher RS accumulated in the rhizome part of *N. nucifera* and *T. angustifolia* (39.34% and 37.19%, respectively). These values are comparable with *C. esculenta* (35.19%) and *Trapa* sp. (36.80%) starches from previous studies [35, 36]. Lower RS was observed in *T. bispinosa* seed with 6.92%, whereas *S. sagittifolia* corm starch had a comparable value of RS (35.39%) and non-RS (37.37%). Besides, *C. esculenta* starch had significantly higher non-RS compared to others. Generally, high-amylose starch possessed high RS; however, in the present study, the *N. nucifera* seed had higher digestible starch (44.36%) compared to its RS (27.94%). The present study showed that RS was higher mostly in the rhizome part compared to others due to their B-type crystallinity. Densely packed B-type and CB-type crystallinity patterns are poorly susceptible to hydrolysis containing higher RS compared to A-type [37]. They further reported that high-amylose starch tends to possess higher RS as studied in cereal starch due to low digestibility. This factor can benefit the consumer interested in a low-carbohydrate dieting regime as slow digestion of RS (digestion occurs after 5-7 hours) can lower insulin and glycemic response and accelerate the satiation level [38]. The variations in resistant starch content between starches of the same species with previous studies (Table 3) were due not only to the chemical parameters (e.g., amylose and phosphorus contents) but also to the physical and structural characteristics such as shape and of granule, molecular interaction and crystallinity pattern of each plant [39]. Also, RS brings benefits in the functional properties of starch in terms of granules swelling, viscosity paste, the formation of gel, and water-binding capacity [37]. The fine, white, and tasteless characteristic of RS particles and its crispness properties make it easy to process and improve the texture, appearance, and mouthfeel in the final product. Silvi et al. [40] reported that RS is an excellent dietary supplement because it can increase good prebiotic microorganisms and reduce the population of harmless Enterobacteria in the colon. However, excessive consumption of RS, mainly the raw starch, will have adverse effects on gastrointestinal performance such as bloating, flatulence, and borborygmi (stomach rumble due to fluid and gas movement in the intestines) and diarrhea. According to Food and Agriculture Organization (FAO) and World Health Organization (WHO), adequate intakes for total fiber are differed based on gender, with 38 g for men, while 25 g for women [41]. Baghurst et al. [42] reported total intakes of approximately 30-40 g per day of RS consumed in developing countries. A 20 g of RS per day consumption is the recommendation by Australia's Commonwealth Scientific and Industrial Research Organization (CSIRO). This amount is much higher than those in Europe of 3-6 g/day due to accessibility in bread and cereals [42]

### 3.4. Starch Crystallinity

**Figure 2:**
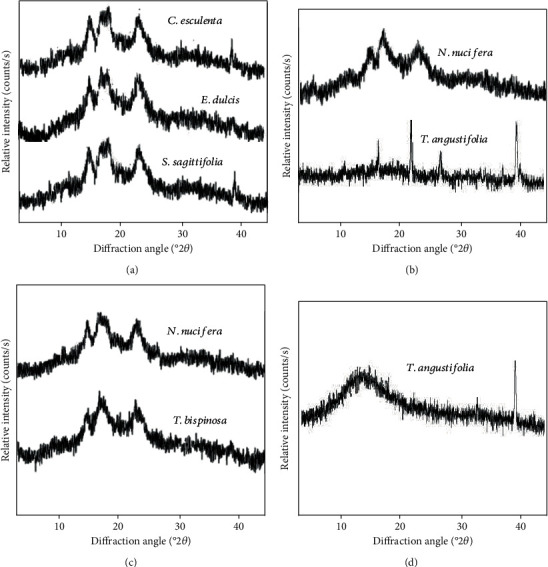
XRD pattern of aquatic macrophytes based on their parts. (a) Corm, (b) rhizome, (c) seed, and (d) pollen samples.

The XRD profiles of aquatic macrophytes are presented in Figure 2, which exhibited well-resolved and intense peaks based on their parts. There are three types of XRD patterns categorized based on their absence of a peak at a certain different angle. The A-type X-ray pattern is characterized with a strong double peak at 2*θ* ≈ 17°and 18°, and strong diffraction peak at 2*θ* ≈ 15°and 23°, whereas the B-type pattern showed pronounced peak diffraction peak at 2*θ* ≈ 15° and 17°, as well broad diffraction peak at 2*θ* ≈ 23°, with smalls peak around 15°, 20°, 22°, and 24° 2*θ* [22, 46]. The C-type is a mixture of A-type and B-type which can be further categorized to C_A_-type if closer to A-type and C_B_-type if closer to B-type, or prominent C-type if the proportion of A- and B-type allomorphs are equal [46]. According to Kim et al. [47], the X-ray diffraction pattern indicates the parallel stranded double helices where A-type structure is more compact than the looser crystallinity of B-type with a more open structure. Furthermore, seed starches generally possessed A-type pattern, whereas tuber and high-amylose cereal starches mostly showed B-type patterns [48].

Based on the present study, corm samples and seed of *N. nucifera* displayed A-type with the presence of peaks intensity at 2*θ* ≈ 15°and 23° and strong doublet around 17°and 18° 2*θ* (Table 4), consistent with the previous studies of arrowhead [49], Chinese water chestnut [50], taro [51], and lotus seed [48]. *Trapa bispinosa* exhibits A-type characteristic with small peak intensity at 2*θ* ≈ 20° (24.73%) which results in C_A_-type similar to the water caltrop species (*T. bispinosa*, *T. quadrispinosa*, *T. pseudoinisa*, and *T. taiwanensis*) of previous studies [12, 52–54]. Furthermore, all the rhizome parts and *T. angustifolia* pollen starch exhibited C_B_-type crystallinity with peaks at 5°, 20°, and 24° 2*θ* (5.96). Both B- [55, 56] (additional) and C-type [48, 57] XRD patterns in lotus rhizome suggest that temperature and other factors influence the crystalline order of lotus rhizome [56]. The XRD pattern of cattail rhizome is contrasting with previous literature in which there was the presence of A- and B-type XRD; however, higher B-type allomorphs were reported compared to A-type [14]. The XRD pattern of rhizome and pollen of cattails in the present study is poor based on their peak intensity values, caused by other impurities in starch granules.

A relatively higher crystallinity was in *C. esculenta* corm (36.91%), followed by *T. bispinosa* seed (35.59%). There was a lower relative crystallinity in *T. angustifolia* pollen (15.27%) and rhizome (15.56%). The value of relative crystallinity is directly proportional to the amylose content. It expends higher relative crystallinity in the waxy starch of *C. esculenta* corm and *T. bispinosa*. The lower amylose content reduced the disruption of crystalline nature and the amorphous region within the starch granules [57].

### 3.5. Thermal Properties

**Figure 3:**
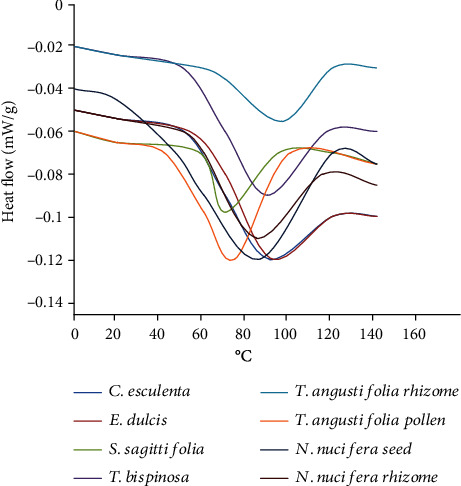
DSC curves of aquatic macrophytes starches.

Table 5 shows a summary of the thermal properties results for the starch samples. The starches gave well-defined single endotherms, where the gelatinization onset (To), peak (*Tp*), and conclusion (*Tc*) temperatures of the aquatic macrophytes starches ranged 41.62-58.74°C, 74.62-96.02°C, and 99.89-119.59°C, respectively (Figure 3). In contrast, gelatinization enthalpies (Δ*H*) ranged 3.85-19.95 J g^−1^. Higher To and *Tp* were observed for *T. angustifolia* rhizome starch (58.73°C and 96.02°C), while the lower results were obtained for *T. angustifolia* pollen starch (41.63°C and 74.62°C), respectively. The higher *Tc* value was observed for *S. sagittifolia* corm (119.58°C), *T. angustifolia* rhizome (119.56°C), and *C. esculenta* corm (119.54°C) starch, while the lower *Tc* was for *T. angustifolia* pollen starch (99.89°C). On average, the gelatinization temperature range (*Tr*) and PHI values of the native starches' samples were 36.45°C and 0.61, respectively. Temperature ranges from 39.57°C in *T. bispinosa* seed to 39.41°C in *N. nucifera* seed. The starch granules size, crystallinity degree, amylose-amylopectin ratio, and the amylopectin branch's size influence the To and *Tp* values [50]. In this present study, a higher To and *Tp* were in *T. angustifolia* rhizome, 58.73°C and 96.02°C, respectively. The formation of amylose and lipid complex in starch is insoluble in water. A higher temperature is required to dissociate the formation and increase the individual granules' swelling to diffuse out the amylose from swollen granules [58]. On the other hand, the *Tc* was observed higher in most of the smaller granules of *E. dulcis* corm (119.58°C), *T. angustifolia* rhizome (119.56°C), and *C. esculenta* corm (119.54°C). The small-sized granules contain a more significant number of granules per unit weight, which needed higher temperatures to disintegrate the starch particles into paste [9]. Tester and Morrison [59] explained that gelatinization enthalpy (Δ*H*) reflects the loss of molecular order within the granules. The high Δ*H* lowers the starch crystallinity degree (quality and quantity of crystallites). PHI measured the gelatinization uniformity. The Δ*H* is generally associated with double helices of amylopectin chains. A higher amylopectin content result in higher Δ*H*. However, the present study showed that higher Δ*H* and PHI were in starch with higher amylose content such as *N. nucifera* seed (19.93 J g^−1^, 1.01) and *T. angustifolia* pollen (18.66 J g^−1^, 1.13), respectively. A higher enthalpy transition is needed to uncoil the long chain of amylopectin possessed by those starches. The present study showed a broad range of temperature *Tr* (33.00-39.57°C), which were comparable with the previous study of commercial starches such as corn (32.2°C), sweet potato (38.94°C), and potato (39.87°C) [60–62]. In addition, Wani et al. [63] also reported a higher endothermic curve in *S. sagittifolia* corm starch for *Tp* (101.5°C) and *Tc* (116.30) values. According to Yu and Christie [64], broadening or overlapping between two neighbouring peaks may be due to sampling mass higher than 5 mg as it lowered the resolution. Higher temperatures may also cause by decreasing water/starch ratio [65].

### 3.6. Rheological Behaviour

Rheology is the study of the deformation and flow of food properties. According to Nurul et al. [66], starch expressed unique viscosity behaviour depending on the changes in temperature, concentration, and shear rate. Present studies showed that the viscosity decreased as the shear stress decreased as characterized by non-Newtonian fluids. *Colocasia esculenta* corm (46.2 mPa s) and *N. nucifera* rhizome starch (40.9 mPa s) possess higher viscosity in the first cycle with the shear stress of 71.71 (Pa) and 63.35 (Pa), respectively (Table 6). Both possess a waxy structure characterized by amylopectin, which yielded larger molecules than amylose [20]. On the other hand, lower viscosity was observed in the *T. angustifolia* pollen (3.2, 3.0, and 2.9 mPa s) due to low-amylose content, as it inhibits the swelling of granules, thus lowering the viscosity [67]. Also, *T. bispinosa* seed possesses the lowest viscosity and shear stress (8.8 mPa s/13.72 Pa) despite having high amylopectin content. According to Singh et al. [58], higher phosphate monoesters in water caltrop may associate with amylopectin hence increase the starch viscosity while phospholipids caused opaque and decrease the viscosity pastes. The author also mentioned that starch's protein content is contradictory with peak viscosity and correlated with pasting temperature. Thus, the high phosphorus and protein content of *T. bispinosa* seed starch contribute to its low viscosity.

## 4. Conclusions

Starches from *T. angustifolia* (cattails) and *S. sagittifolia* (arrowheads) have comparable potentials to commercial aquatic macrophyte starches from *N. nucifera* (lotus), *E. dulcis* (Chinese water chestnut), *T. bispinosa* (water caltrop), and *C. esculenta* (taro) in food formulation. Although the least starch was isolated from *T. angustifolia* pollen, its higher amylose content (36.47%) provides excellent human digestive tract benefits. Higher RS content was in tuber parts, i.e., rhizome and corm, than in the seed and pollen of freshwater macrophytes. Lower RS was found in *N. nucifera* seed starch (27.94%) compared to others despite having higher amylose. Starch isolation procedure of *T. angustifolia* needs to be improvised, especially on the starch washing to prevent the impurities affecting the functional properties. Simultaneously, the procedure also must be cost-effective and efficient to be commercialized in the large-scale industry. This functionality of the native starches can later be modified and improved physically, chemically, and enzymatically which enhanced its properties and to produce novel molecules and hence can be applied in a broad range of food and nonfood applications. The starch properties in aquatic macrophytes studies showed equally good potential as the commercial starches in starch-based products.

## Figures and Tables

**Table 1:** Starch granule characteristics and size, based on commercial starch by Pomeranz [19].

Species	Part	Granules size (*μ*m)	The shape of starch granule	Hilum
Small starch granule (3-8 *μ*m)				
*C. esculenta*	Corm	2.95 ± 0.47^de^	Polygonal, irregular, and oval	Centric
*E. dulcis*	Corm	6.74 ± 0.56^bc^	Big elongated granule, small oval granule, and spherical with smooth surfaces	Centric
*S. sagittifolia*	Corm	7.53 ± 0.46^b^	Round and oval with smooth surfaces	Centric and eccentric
*T. bispinosa*	Seed	7.90 ± 0.44^b^	Elongated with smooth surfaces	Centric
*T. angustifolia*	Rhizome	4.69 ± 0.46^cd^	Round and oval with smooth surfaces	Centric
*T. angustifolia*	Pollen	2.09 ± 1.16^e^	Polygonal, irregular, and oval	Centric
Medium-sized starch granule (10-25 *μ*m)				
*N. nucifera*	Seed	9.22 ± 0.92^b^	Oval and ellipsoidal with a smooth surface	Centric
Large starch granule (15-100 *μ*m)				
*N. nucifera*	Rhizome	20.96 ± 1.86^a^	Small oval granules, large longitudinal, rod-shaped granules	Centric and eccentric

Data are mean values of *n* = 30 determination ± standard error, and different superscripts (a>b>c>d>e) are significantly different (DMRT, *p* < 0.05).

**Table 2:** Total starch, proximate compositions and phosphorus content of freshwater macrophytes starches.

Species	Total starch (%)	Amylose (%)	Moisture (%)	Protein (%)	Lipid (%)	Phosphorus (ppm)
*C. esculenta* corm	82.35 ± 1.18^b^	10.61 ± 0.13^e^	10.70 ± 0.08^cd^	5.67 ± 0.03^d^	0.49 ± 0.08^d^	0.04 ± 0.01^bc^
*E. dulcis* corm	90.87 ± 1.56^a^	11.48 ± 0.60^e^	17.23 ± 0.26^a^	4.39 ± 0.19^e^	0.24 ± 0.15^d^	0.13 ± 0.01^a^
*S. sagittifolia* corm	71.71 ± 1.83^c^	19.80 ± 0.47^c^	11.91 ± 0.25^bc^	3.06 ± 0.02^f^	0.61 ± 0.21^d^	0.02 ± 0.00^c^
*T. bispinosa* seed	49.56 ± 0.61^e^	8.83 ± 0.10^f^	14.43 ± 0.16^ef^	26.47 ± 0.83^a^	1.13 ± 0.13^c^	0.08 ± 0.01^abc^
*T. angustifolia* rhizome	66.02 ± 1.68^d^	13.51 ± 0.11^d^	11.86 ± 0.16^bc^	4.86 ± 0.51^de^	3.87 ± 0.16^a^	0.09 ± 0.06^abc^
*T. angustifolia* pollen	7.02 ± 0.05^f^	36.47 ± 0.49^b^	8.91 ± 0.57^f^	15.70 ± 0.14^b^	3.23 ± 0.12^b^	0.10 ± 0.01^ab^
*N. nucifera* seed	67.69 ± 0.13^d^	71.45 ± 1.04^a^	12.93 ± 0.13^b^	7.24 ± 0.20^c^	2.96 ± 0.24^b^	0.05 ± 0.01^bc^
*N. nucifera* rhizome	66.00 ± 1.68^d^	7.63 ± 0.21^f^	11.84 ± 0.17^bc^	7.96 ± 0.05^c^	0.42 ± 0.12^d^	0.05 ± 0.01^bc^

Data are mean values of *n* = 3 determination ± standard error, and different superscripts (a>b>c>d>e>f>g) are significantly different (DMRT, *p* < 0.05).

**Table 3:** Resistant and nonresistant starch of the present study in comparison with other previous studies.

Species/part	Resistant starch (%)	Nonresistant starch (%)	References
*C. esculenta* corm	18.35 ± 0.47	64.87 ± 2.63	Present study
*E. dulcis* corm	37.41 ± 0.21^a^	45.29 ± 1.25^c^	Present study
*S. sagittifolia* corm	35.09 ± 0.53^b^	37.37 ± 0.98^d^	Present study
*T. bispinosa* seed	6.92 ± 1.58^f^	56.94 ± 1.35^b^	Present study
*T. angustifolia* rhizome	37.19 ± 1.05^ab^	21.10 ± 0.76^f^	Present study
*T. angustifolia* pollen	9.65 ± 0.08^e^	3.52 ± 0.22^g^	Present study
*N. nucifera* seed	27.94 ± 0.37^c^	44.36 ± 0.34^c^	Present study
*N. nucifera* rhizome	39.34 ± 0.06^a^	27.69 ± 0.82^e^	Present study

Previous studies			
Aquatic macrophytes starch			
*C. esculenta*	1.99	—	[43]
*C. esculenta*	2.11	—	[43]
*C. esculenta*	2.26	—	[43].
*C. esculenta*	35.19	35.73	[44]
*C. esculenta*	44.10	—	[36]
*C. esculenta*	27.50	—	[36]
*C. esculenta*	4.12	59.61	[45]
*Trapa* sp.	41.50	—	[36]
*Trapa* sp.	36.80	—	[36]
*E. dulcis*	8.05	—	[36]
*N. nucifera* rhizome	2.10	—	[36]
*N. nucifera* seed	19.70	—	[36]

Commercialized starch			
*Dioscorea alata*	22.48	18.85	[44]
*Ipomea batatas*	0.97	75.55	[44]
*I. batatas*	28.90	—	[36]
*I. batatas*	3.19	49.35	[45]
*Zea mays*	7.83	—	[36]
*Solanum tuberosum*	79.30	—	[36]
*Manihot esculenta*	80.80		[36]
*M. esculenta*	9.69	55.99	[45]
*Oryza sativa* (waxy)	2.72	72.04	[45]

Data are mean values of *n* = 3 determination ± standard error, and different superscripts (a>b>c>d>e>f) are significantly different (DMRT, *p* < 0.05).

**Table 4:** Degree of crystallinity and X-ray pattern of native starch extracted from aquatic macrophytes.

Species/part	X-ray pattern	Relative crystallinity (%)	Different peak intensity at 2*θ* value (%)							
			5°	15°	17°	18°	20°	22°	23°	24°
*C. esculenta* corm	A	36.91	—	31.35	29.10	31.77	—	—	21.16	—
*E. dulcis* corm	A	21.34	—	29.05	36.50	28.87	—	—	32.82	—
*S. sagittifolia* corm	A	21.28	—	27.37	30.15	22.83	—	—	18.66	—
*T. bispinosa* seed	C_A_	35.59	—	34.86	57.16	47.52	24.73	—	33.72	—
*T. angustifolia* rhizome	C_B_	15.56	—	3.07	3.96	—	15.80	—	—	2.77
*T. angustifolia* pollen	C_B_	15.27	1.99	3.05	—	—	—	—	—	—
*N. nucifera* seed	A	29.20	—	27.29	36.97	21.95	—	—	20.18	—
*N. nucifera* rhizome	C_B_	24.20	5.96	16.71	34.16	—	—	—	18.24	—

**Table 5:** Thermal properties of native starch extracted from aquatic macrophytes.

Species/part	*T*_*o*_ (°C)	*T*_*p*_ (°C)	*T*_*c*_ (°C)	Δ*H*_gel_ (J g^−1^)	PHI	*T*_*r*_ (°C)
*C. esculenta* corm	50.91 ± 0.09^d^	89.85 ± 0.02^d^	119.54 ± 0.03^a^	13.73 ± 0.00^c^	0.71 ± 0.0^d^	38.94 ± 0.07^c^
*E. dulcis* corm	53.14 ± 0.04^c^	91.35 ± 0.04^c^	119.58 ± 0.01^a^	12.81 ± 0.00^f^	0.67 ± 0.00^e^	38.21 ± 0.03^d^
*S. sagittifolia* corm	58.17 ± 0.02^b^	94.45 ± 0.02^b^	119.34 ± 0.04^c^	7.31 ± 0.01^g^	0.40 ± 0.00^g^	36.28 ± 0.01^f^
*T. bispinosa* seed	50.26 ± 0.03^e^	89.83 ± 0.04^d^	116.96 ± 0.03^d^	13.15 ± 0.02^d^	0.66 ± 0.00^f^	39.57 ± 0.03^a^
*T. angustifolia* rhizome	58.73 ± 0.01^a^	96.02 ± 0.00^a^	119.56 ± 0.00^a^	3.85 ± 0.00^h^	0.21 ± 0.00^h^	37.28 ± 0.00^e^
*T. angustifolia* pollen	41.63 ± 0.00^h^	74.62 ± 0.00^g^	99.89 ± 0.00^f^	18.66 ± 0.01^b^	1.13 ± 0.00^a^	33.00 ± 0.01^h^
*N. nucifera* seed	46.85 ± 0.04^g^	86.26 ± 0.04^e^	119.45 ± 0.03^b^	19.93 ± 0.02^a^	1.01 ± 0.01^b^	39.41 ± 0.05^b^
*N. nucifera* rhizome	50.13 ± 0.03^f^	84.07 ± 0.02^f^	115.15 ± 0.05^e^	13.12 ± 0.02^e^	0.77 ± 0.00^c^	33.94 ± 0.03^g^

Data are mean values of *n* = 3 determination ± standard error, and different superscripts (a>b>c>d>e>f>g>h>i>j) are significantly different (DMRT, *p* < 0.05).

**Table 6:** Rheological behaviour of freshwater macrophytes starches.

Species/part	Temp. (°C)	Viscosity (mPa s)/shear stress (Pa)		
		1st rotation	2nd rotation	3rd rotation
*C. esculenta* corm	32.9	46.2/71.71	42.7/66.25	40.7/63.1
*E. dulcis* corm	32.7	27.6/42.86	26/40.36	25.4/38.21
*S. sagittifolia* corm	32.7	28.4/44.02	26.5/41.16	24.3/38.90
*T. bispinosa* seed	32.4	8.8/13.72	8.8/13.58	8.0/13.49
*T. angustifolia* rhizome	32.6	8.0/12.37	8.1/12.89	8.0/12.89
*T. angustifolia* pollen	32.4	3.2/4.93	3.0/4.68	2.9/4.45
*N. nucifera* seed	33.0	28.0/43.45	25.7/39.83	23.8/35.4
*N. nucifera* rhizome	33.0	40.9/63.35	37.2/57.72	34.8/51.2

## Data Availability

The data used to support the findings of this study are included in the article.
